# The Role of Laser in the Treatment of Cervical Dentin Hypersensitivity

**DOI:** 10.7759/cureus.82956

**Published:** 2025-04-24

**Authors:** Mohamed Rahhali, Nissrin Bassim

**Affiliations:** 1 Conservative Dentistry and Endodontics, Faculty of Dentistry, Mohammed V University, Rabat, MAR; 2 Conservative Dentistry and Endodontics, Mohammed V Military Hospital of Rabat, Rabat, MAR

**Keywords:** co2 laser, dentin hypersensitivity, laser-therapy, nd :yag, soft laser

## Abstract

Cervical dentin hypersensitivity (CDH) is a common condition characterized by sharp, transient pain caused by exposed dentinal tubules, often resulting from periodontal disease or other underlying factors. Conventional home and in-office desensitization treatments may fail to provide adequate relief in persistent cases. This case study presents the successful management of CDH using a non-invasive laser-assisted approach. Nd:YAG laser therapy was applied to affected teeth, effectively sealing dentinal tubules and modulating nerve conduction, leading to immediate and complete pain resolution. The treatment outcome underscores the potential of laser therapy as an alternative desensitization strategy, offering rapid and efficient symptom relief. Laser technology, with its ability to promote tissue stability and reduce hypersensitivity, presents a promising option for enhancing patient comfort and long-term clinical success.

## Introduction

Cervical dentin hypersensitivity (CDH) is characterized by intense but brief pain in response to various thermal, tactile, osmotic, or chemical stimuli, typically occurring at the cervical level of the tooth. This pathognomonic pain cannot be attributed to any other dental abnormality, disease, or restorative treatment. CDH results from the exposure of dentinal tubules due to gingival recession, cementum loss, or non-carious cervical lesions, which may arise from periodontal disease, excessive root planing, or erosive and abrasive processes. Although it is a common issue in dental practice, it remains underdiagnosed and often inadequately managed [1].

The hydrodynamic theory proposed by Brännström remains the most widely accepted explanation for CDH, suggesting that external stimuli induce fluid movement within exposed dentinal tubules, causing pressure variations that activate nerve endings at the pulp-dentin junction, resulting in sharp and transient pain [2]. Other mechanisms, such as the potential role of odontoblasts in pain signal transmission, have been suggested but lack strong clinical validation.

The prevalence of CDH varies considerably, affecting between 3% and 57% of adults, depending on the population studied and the method used for evaluation. Most studies indicate that it is more prevalent among women [3]. Given its multifactorial etiology and the variability in patient response, numerous therapeutic approaches have been proposed. Laser therapy, particularly the Nd:YAG laser, has emerged as an effective option due to its ability to occlude dentinal tubules and reduce pulpal nerve sensitivity. While its use is well documented in the literature [4], this case report aims to provide a detailed clinical illustration of its application in a patient who did not respond to conventional treatments. The objective is not to introduce a novel technique but to reinforce current clinical practices by sharing protocol details, clinical reasoning, and short-term outcomes.

## Case presentation

A 40-year-old male patient presented to the Department of Conservative Dentistry at Mohammed V Military Hospital in Rabat, Morocco, with a chief complaint of persistent CDH affecting the right maxillary premolar (#15) and molar (#16). The patient described discomfort triggered by thermal stimuli, particularly when consuming cold or hot foods and during routine oral hygiene. His medical history was unremarkable, with no systemic conditions or ongoing medications. However, he had a history of periodontal disease, which had been previously treated and stabilized.

**Figure 1:**
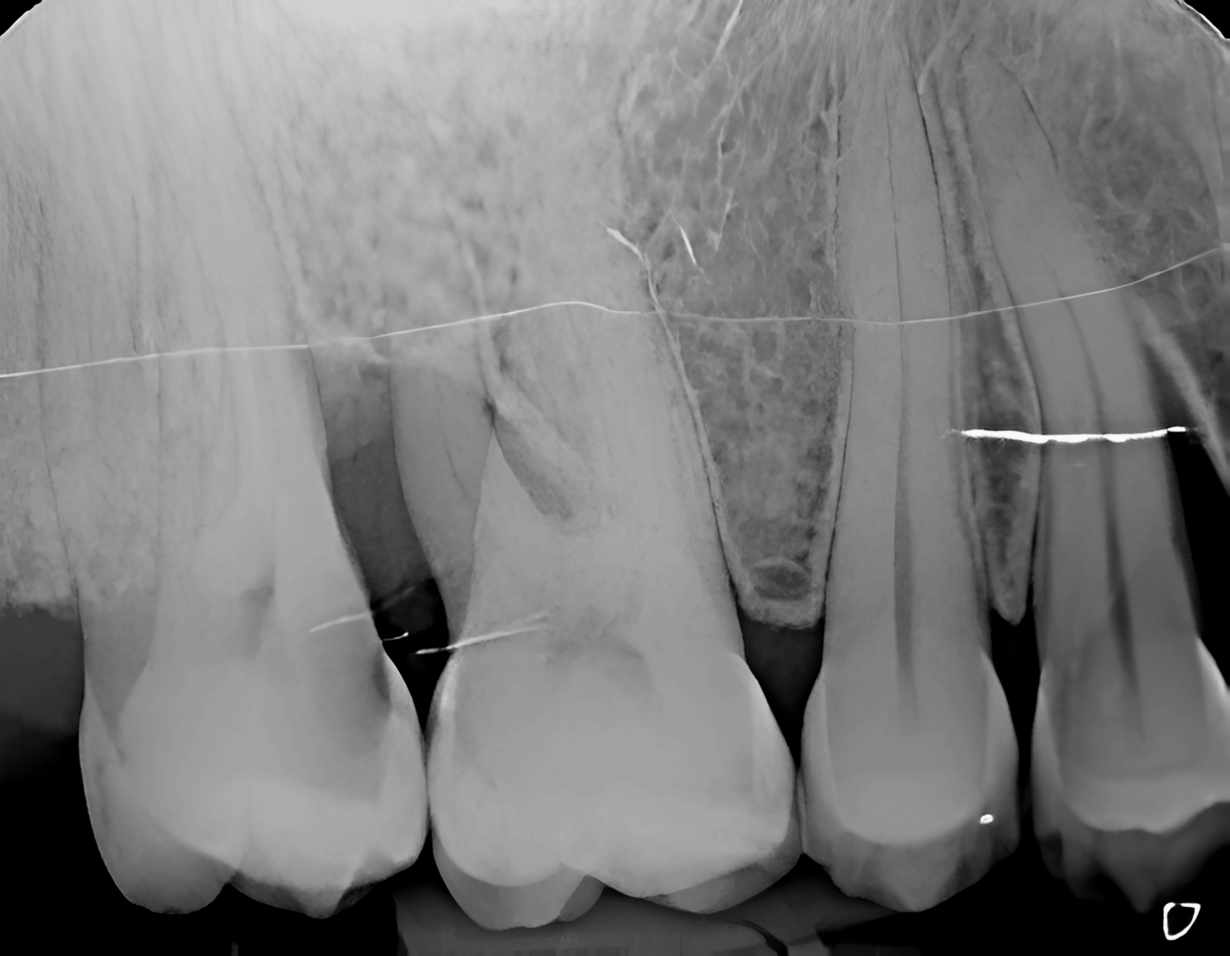
Periapical radiograph of teeth #15 and #16

Upon clinical examination, Miller class II gingival recession was observed on teeth #15 and #16, exposing the root surfaces. The cold test elicited a positive response, reproducing the patient’s symptoms and confirming the diagnosis of CDH at teeth #15 and #16. A periapical radiograph revealed no radiolucency suggestive of caries or other pathological findings (Figure 1).

As a first-line treatment, the patient was prescribed a desensitizing toothpaste containing 0.32% sodium fluoride and 5% potassium nitrate (Sensi Kin, KIN, Spain), along with modifications to his brushing technique. After two weeks, the patient reported persistent sensitivity with no significant improvement.

To enhance desensitization, a fluoride varnish (MI Varnish®, GC, Japan), containing sodium fluoride combined with calcium phosphate, was applied once per week for two consecutive weeks. However, the patient only experienced a slight reduction in sensitivity, which was not clinically significant. Given the persistence of symptoms, Nd:YAG laser therapy was proposed as an alternative desensitization approach.

**Figure 2:**
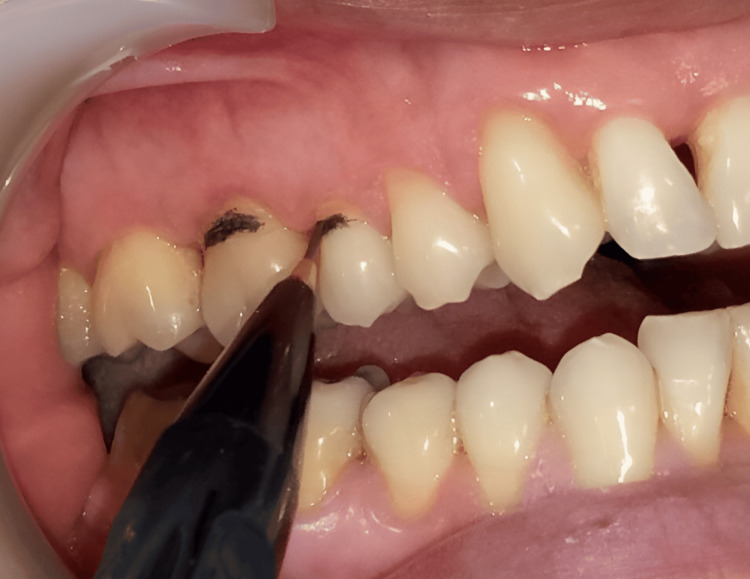
Graphite coating of the dentin surface of teeth #15 and #16 using the charcoal pencil marking

Before initiating the laser procedure, the exposed dentin surfaces were coated with a thin layer of graphite (Figure 2) to enhance absorption of laser light and improve treatment efficiency. The Nd:YAG laser (LightWalker, Fotona, Slovenia) was set to a wavelength of 1064 nm, with an energy output ranging from 0.5 W to 0.75 W, operating in continuous mode.

**Figure 3:**
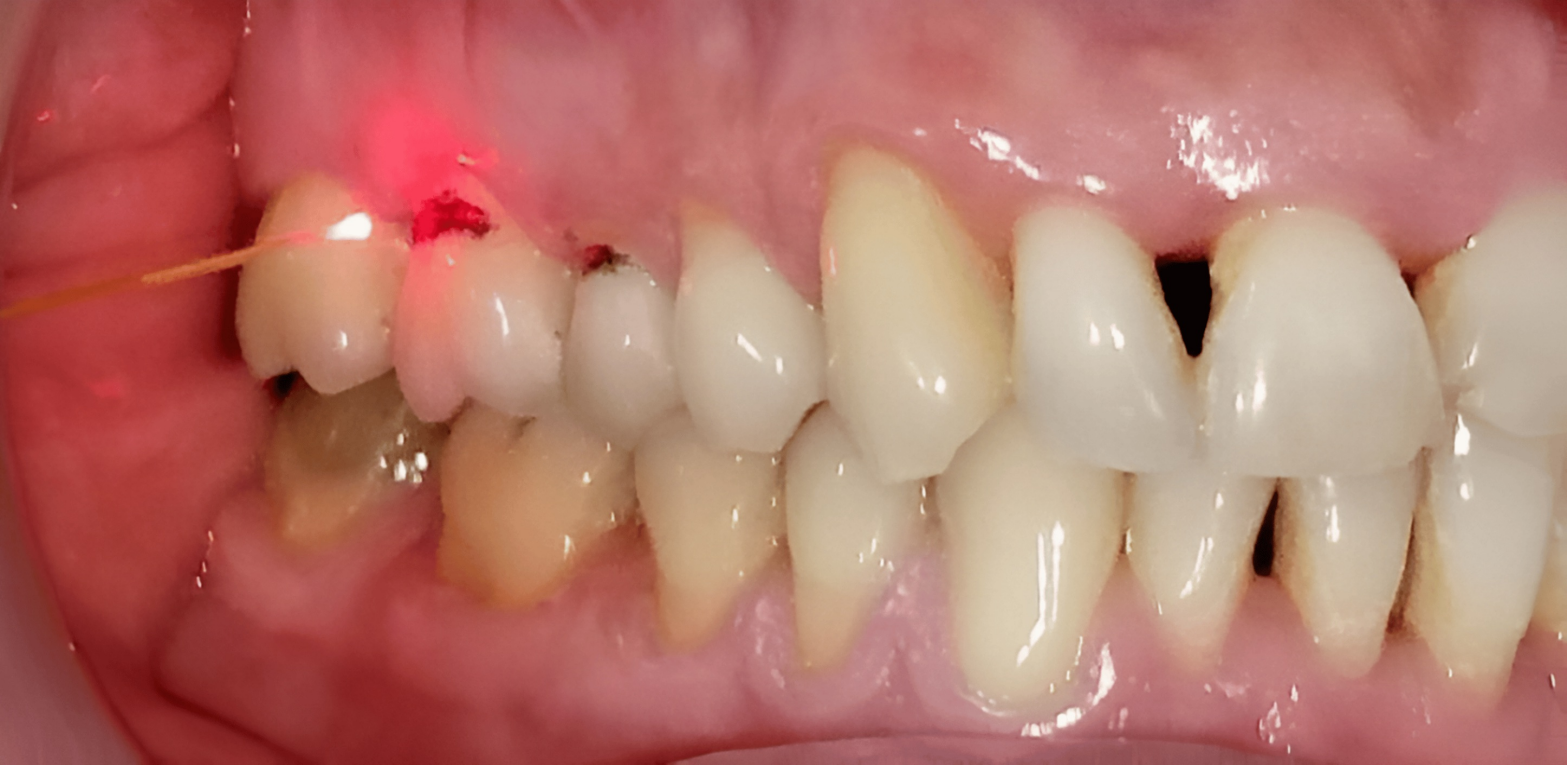
Sweeping with the Nd:YAG laser on teeth #15 and #16

The laser application used a 300 µm fiber optic tip, which was moved in a tangential sweeping motion over the exposed dentinal surface (Figure 3). Each affected tooth (#15 and #16) received two applications, each lasting 15 seconds, to ensure uniform coverage of the hypersensitive area while avoiding excessive heat buildup.

**Figure 4:**
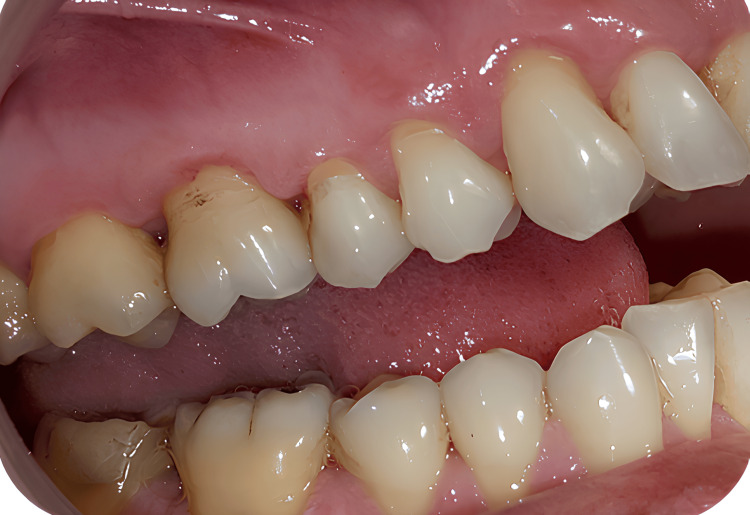
Surface appearance after Nd:YAG laser application on teeth #15 and #16

Throughout the procedure, the patient and operator wore protective eyewear to prevent inadvertent exposure to laser emissions. No anesthesia was required, as the laser application was well tolerated. Figure 4 shows the surface appearance following Nd:YAG laser application on teeth #15 and #16.

The immediate post-treatment response showed a complete resolution of hypersensitivity. The patient was advised to continue using a desensitizing toothpaste as part of long-term maintenance.

Immediately after the procedure, the patient reported complete pain relief, with no discomfort upon air stimulation or cold testing. He was advised to continue using a desensitizing toothpaste for maintenance. At the six-month follow-up, the patient remained asymptomatic, confirming the effectiveness of Nd:YAG laser therapy in sealing dentinal tubules and reducing hypersensitivity. Regular follow-ups were scheduled for long-term monitoring.

## Discussion

CDH is a condition caused by the exposure of dentinal tubules due to gingival recession, cementum loss, or non-carious cervical lesions. It is particularly prevalent among young adults and is often associated with poor dietary habits, aggressive brushing techniques, and excessive use of whitening products. The most widely accepted explanation for CDH remains Brännström’s hydrodynamic theory, which attributes pain to fluid movement within exposed dentinal tubules, leading to pressure variations that stimulate nerve endings at the pulp-dentin junction. Other mechanisms, such as odontoblast-mediated pain transmission, have been proposed but lack strong clinical evidence [1,3].

Given the variable nature of symptomatology and individual pain perception, CDH is a diagnosis of exclusion. It must be distinguished from carious lesions, defective restorations, cracked teeth, or post-whitening sensitivity. Treatment typically follows a progressive approach, beginning with conservative measures such as dietary and oral hygiene modifications, followed by desensitizing agents in toothpaste, gels, or mouthwashes. These products, which contain fluoride, nano-hydroxyapatite, arginine-calcium carbonate, potassium oxalate, and strontium-based compounds, effectively reduce dentin sensitivity [5-8]. When symptoms persist, in-office treatments become necessary.

Among professional interventions, tubule-occluding agents such as fluoride varnishes, potassium oxalate, and casein phosphopeptide-amorphous calcium phosphate (CPP-ACP)-based formulations help reinforce dentin and decrease permeability. Adhesive primers and infiltration resins provide long-term relief by forming a protective hybrid layer [9-12]. Laser therapy, an emerging approach, achieves desensitization through two primary mechanisms: nerve modulation, which reduces pain perception, and tubule sealing, which limits fluid movement. Nd:YAG, CO₂, and low-level laser therapy have demonstrated high efficacy in managing CDH. However, Er:YAG lasers remain contraindicated due to their dentin-ablative properties, which can exacerbate sensitivity [13].

The effectiveness of Nd:YAG laser therapy in treating CDH has been extensively studied, with reported success rates ranging between 85% and 100%. It has been shown to provide significant pain reduction when used in continuous mode at controlled energy levels (0.5-0.75 W), which helps ensure minimal pulpal damage. However, its long-term effects remain a subject of debate [14]. Some studies have found Nd:YAG therapy to be comparable to alternative treatments such as calcium sodium phosphosilicate paste (NovaMin®) and MI Varnish™ (CPP-ACP), with no statistically significant difference in long-term desensitization outcomes. A comparative study even suggested that MI Varnish™ might offer better tactile sensitivity reduction over six months. These findings are consistent with the results observed in this case, confirming that while Nd:YAG laser therapy is highly effective, alternative treatments offer comparable and cost-effective results. Consequently, laser therapy should be considered as one of several viable treatment options rather than the definitive solution for all CDH cases [15,16].

The duration of desensitization achieved with Nd:YAG and CO_2_ lasers typically ranges from six months to one year. Unlike Nd:YAG lasers, CO₂ laser therapy can result in surface discoloration, which may require additional polishing for aesthetic reasons. Additional restorative interventions, such as glass ionomer cement or composite resin restorations, can seal exposed dentin for severe cases of hypersensitivity. Mucogingival grafting may also be required to cover receded areas [17].

Considering the breadth of evidence supporting various desensitization approaches, Nd:YAG laser therapy should not be the definitive treatment for all CDH cases. Instead, treatment selection should be guided by patient-specific factors, including the severity of sensitivity, cost, accessibility to laser technology, and the need for reapplication. Incorporating alternative desensitizing agents, such as CPP-ACP varnishes and calcium sodium phosphosilicate pastes, into clinical practice expands the range of effective options for managing CDH while ensuring similar therapeutic outcomes.

This case report is limited by its single-patient design, which restricts the generalizability of the findings. Additionally, the short follow-up period prevents any conclusions about the long-term effectiveness and recurrence of symptoms. Future studies should include larger patient cohorts with extended follow-up to better evaluate the comparative durability of Nd:YAG laser therapy versus other desensitization techniques. Randomized controlled trials and standardized outcome measures would also be beneficial in establishing clearer clinical guidelines for using lasers in managing CDH.

## Conclusions

CDH significantly affects patients’ quality of life and requires a preventive, personalized treatment strategy. While Nd:YAG laser therapy offers rapid symptom relief, its effects are often temporary and associated with higher costs. Given the comparable clinical efficacy of alternative desensitizing agents, treatment choice should be guided by factors such as symptom severity, accessibility of care, cost considerations, and the likelihood of reapplication, ensuring an approach that is both effective and practical for long-term management.
